# Lipid safety of tenofovir alafenamide during 96-week treatment in treatment-naive chronic hepatitis B patients

**DOI:** 10.3389/fmed.2024.1399665

**Published:** 2024-06-04

**Authors:** Wenjuan Zhao, Yi Liu, Mengdi Zhang, Zixin Cui, Zhan Qu, Yiyang Li, Meijuan Wan, Wen Wang, Yunru Chen, Lei Shi, Jianzhou Li, Feng Ye

**Affiliations:** ^1^Department of Infectious Diseases, The First Affiliated Hospital of Xian Jiaotong University, Xi’an, China; ^2^Department of Nutrition, Xian Jiaotong University, Xi’an, China

**Keywords:** tenofovir alafenamide, lipid safety, chronic hepatitis B, 96-week, treatment

## Abstract

**Background:**

This study was aimed at investigating the dynamics of lipids and the effect of TAF on the lipid profile of patients including fatty liver disease in CHB patients.

**Methods:**

The data of TC, LDL-c, HDL-c, TG, and TC/HDL ratio were collected at baseline, 24 weeks, 48 weeks, 72 weeks, and 96 weeks. CHB patients with fatty liver at baseline were further analyzed in a subgroup.

**Results:**

A total of 137 CHB patients treated with TAF were enrolled in this study. During 96 weeks of TAF treatment, there was no significant change in TC, LDL-c, HDL-c, and TG level (*P* > 0.05). The TC/HDL-c ratio was increased with no significant change (+0.24, *P* > 0.05). In CHB patients with fatty liver (*n* = 48), TC, LDL-c, and TC/HDL-c ratio increased gradually during TAF treatment, TG levels increased to 146.63 mg/dL at 48 weeks (*P* = 0.057) and then decreased, but there was still no significant change compared with the baseline level by 96 weeks (*P* > 0.05).

**Conclusion:**

TAF treatment had a low effect on the lipid profile of CHB patients over the course of 96 weeks, and it was safe even in patients with fatty liver.

**Clinical trial registration:**

[https://www.chictr.org.cn/showproj.html?proj=65123], identifier [ChiCTR2000041005].

## 1 Introduction

Chronic hepatitis B (CHB) is a systemic infectious disease caused by persistent infection with the hepatitis B virus (HBV) (1). It is estimated that approximately 296 million people worldwide are chronically infected with HBV, which could cause as many as 820,000 deaths annually due to cirrhosis, hepatocellular carcinoma, and other complications resulting from the progression of CHB (2).

Tenofovir alafenamide (TAF) is a new effective and well-tolerated nucleotide analogue after Tenofovir disoproxil fumarate (TDF) and entecavir (ETV) (3). TAF targets the RNA-dependent DNA polymerase of HBV and exhibits more potent antiviral activity (4). Therefore, TAF, as a drug that inhibits HBV replication, has become the first-line antiviral drugs recommended by major clinical guidelines for the treatment of HBV infection (5).

Antiviral therapy for CHB was considered a long-term treatment (6). This has increased the concern of physicians and patients about drug-related adverse effects. The effect of TAF on lipids has been the focus of recent attention, but the conclusions are controversial. In clinical studies of TAF-containing regimens treated HBV or HIV-infected patients, it was found that there was weight gain and a significant increase in lipid levels during the treatment (7–9). However, there are also studies found that the majority of patients treated with TAF with elevated LDL cholesterol have either a history of dyslipidemia or elevated baseline lipids, or both (10). And Jeong’s study also showed that patients treated with TAF did not elevated lipids compared to HBV-uninfected controls (11).

Meanwhile, CHB combined with nonalcoholic fatty liver disease (NAFLD) is also becoming more common in clinical practice; it is estimated that 25%–30% of CHB patients have combined NAFLD (12). Studies have shown that obesity, high BMI, hyperlipidemia, and type 2 diabetes mellitus increased the risk of comorbidity with NAFLD in CHB patients (13, 14). Therefore, it is necessary to evaluate the lipid safety and the risk of cardiovascular disease during treatment with TAF, especially in patients with CHB combined with NAFLD. Previous studies suggest that long-term antiviral therapy in such patients may increase body fat mass and visceral fat area through virological suppression (15). However, there are a few studies suggesting that TAF-induced dyslipidemia has little effect on the incidence of NAFLD (16).

Therefore, to better understand the changes of lipid levels during TAF treatment, especially in patients with fatty liver, this prospective cohort study aims to investigate the changes in lipid levels after long-term antiviral therapy with TAF in CHB patients and the effects of patients with CHB combined with fatty liver.

## 2 Material and methods

### 2.1 Patients and clinical study design

This was a prospective cohort study conducted in the Department of Infection of the First Affiliated Hospital of Xi’an Jiaotong University. Inclusion criteria were as follows: (1) patients with age > 18 years old; (2) meeting the diagnostic criteria for CHB in the Chinese 2019 Guidelines for the Prevention and Control of Chronic Hepatitis B(HBsAg and/or HBV DNA positive for more than 6 months); (3) receiving TAF therapy (25 mg/dose) for at least 6 months and not receiving other antiretroviral therapy.

Patients who had one of the following criteria were excluded: (1) patients co-infected with HIV or hepatitis C virus; (2) patients with poorly controlled malignancies including hepatocellular carcinoma; (3) patients with a history of allergy to nucleotide analogues (NAs); (4) patients with undergoing lipid-lowering therapy; (5) women who were pregnant or breastfeeding during the treatment; (6) patients with other causes of steatosis such as alcohol consumption, obesity, metabolic diseases, etc. Written informed consents were obtained from all patients in the study.

The study was approved by the Ethics Committee of our hospital (Ethics No. XJTU1AF2020LSL-024) and was registered under the Chinese Clinical Trial Registry (ChiCTR2000041005).

### 2.2 Research methods

At baseline, 24W, 48W, 72W and 96W of TAF treatment, the included patients were examined HBV-DNA load measured with the Roche COBAS^®^ TaqMan^®^ HBV test (Lower limit of detection, 20 IU/mL) and HBsAg quantification by an Elecsys 2010 immunoanalyzer (Roche). The serum low-density lipoprotein-cholesterol (LDL-c), triglyceride (TG), TC, HDL-c levels as well as the TC/HDL ratio, ALT, AST were measured by fully automated biochemistry and the controlled attenuation parameter (CAP) was measured by transient elastography in the Laboratory Department of the hospital. Trends in lipids using TAF 96W were analyzed according to a continuous follow-up cohort.

To investigate the lipid safety of TAF in a special population, stratified analysis was conducted on the effect of TAF on lipid safety according to the presence or absence of comorbid hypercholesterolemia (TC > 5.2 mmol/L or LDL ≥ 2.5 mmol/L) and fatty liver (CAP > 240 dB/m) at baseline.

Factors influencing TAF-related lipid changes in treatment-naive CHB patients were analyzed by linear regression.

### 2.3 Statistical analysis

Descriptive statistics for demographic and clinical variables were expressed as mean ± standard deviation, median (interquartile spacing), count (*n*), and proportion (%). Data comparing changes in lipid levels at different time points before and after TAF treatment were compared using the Student’s t-test for normally distributed continuous variables and the Kruskal-Wallis test for non-normally distributed continuous variables. The chi-square test between multiple groups was used for dichotomous data.

Spearman’s correlation analysis was used to evaluate the relationship between TAF-related lipid changes and age, HBsAg levels, AST, ALT, and CAP values. The main influencing factors of TAF-related lipid changes were also investigated by multiple linear regression analysis. Data analysis was performed using SPSS 26.0 statistical software, and the significance level was set at *p* < 0.05.

All procedures performed in this study were in accordance with the 1964 Helsinki Declaration and its later amendments or comparable ethics standards.

## 3 Results

### 3.1 Patient characteristics

**FIGURE 1:**
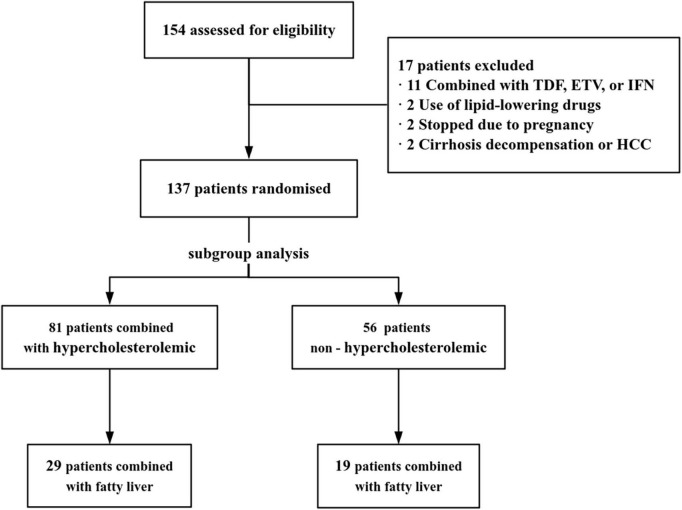
Study flowchart. TDF, tenofovir disoproxil fumarate; ETV, entecavir; IFN, interferon; HCC, hepatocellular carcinoma.

A total of 154 CHB patients treated with TAF were were screened. Among them, 17 were excluded (11 treated with TAF combined with TDF, ETV, or IFN,2 started using lipid-lowering drugs, 2 stopped due to pregnancy, 2 were not available for cirrhosis decompensation or HCC, Figure 1). Baseline characteristics of 137 CHB patients were presented in Table 1. Mean Age was 40.71 ± 10.41 years, 62.04% were male. Subgroup analysis was performed according to whether the baseline lipid were hypercholesterolemia or not. There was no difference between the hypercholesterolemia group (*n* = 81) and the non-hypercholesterolemia group (*n* = 56) in terms of the baseline characteristics (*P* > 0.05), except for the differences in lipid profile and TC/HDL ratio.

**TABLE 1:** Baseline characteristics of the study population and comparison between patients with and without baseline hypercholesterolemia (HC).

Variables	Total	NHC	HC	*P* value
	(*N* = 137)	(*N* = 56)	(*N* = 81)	
Males, *n* (%)	85/137 (62.04)	34/56 (60.71)	51/81 (62.96)	0.79
Females, *n* (%)	52/137 (37.96)	22/56 (39.29)	30/81 (37.04)	
Age (years), mean ± SD	40.71 ± 10.41	40.73 ± 10.5	40.69 ± 10.42	0.982
20-60 yrs (%)	132/137 (94.60)	53/56 (94.64)	79/81 (97.53)	0.376
>60 yrs (%)	5/137 (3.60)	3/56 (5.36)	2/81 (2.47)	
BMI (kg/m^2^), mean ± SD	23.73 ± 2.97	23.84 ± 2.92	23.65 ± 3.05	0.803
**Characteristics of HBV infection**				
HBV DNA (Log10, IU/mL), mean ± SD	5.6 ± 10.51	4.24 ± 2.03	4.21 ± 2.0	0.963
Quantification of HBsAg (Log10, IU/mL), mean ± SD	3.49 ± 0.92	3.51 ± 0.98	3.41 ± 0.86	0.596
HBeAg positive n (%)	73/137 (53.28)	42/81 (51.85)	31/56 (55.36)	0.686
HBeAg negative n (%)	64/137 (46.72)	39/81 (48.15)	25/56 (44.64)	
**Laboratory values**				
Total cholesterol (mg/dL), median (IQR)	168.56 (143.62, 189.82)	140.12 (130.95,156.04)	186.05 (170.81,205.35)	<0.001
LDL cholesterol (mg/dL), median (IQR)	105.16 (84.28,123.33)	79.13 (67.74,87.14)	120.82 (106.73,131.05)	<0.001
HDL cholesterol (mg/dL), median (IQR)	46.40 (39.82,57.99)	44.0 (37.92,51.82)	48.25 (41.5,58.87)	0.051
Triglycerides (mg/dL), median (IQR)	87.71 (65.56,137.77)	77.53 (62.02,109.86)	101.96 (77.13,150.72)	0.094
TC/HDL-C ratio, median (IQR)	3.56 (2.97,4.20)	3.17 (2.64,3.55)	3.79 (3.39,4.53)	<0.001
Blood glucose (mmol/L), mean ± SD	4.92 ± 0.84	4.87 ± 0.86	4.95 ± 0.84	0.619
Phospheremia (mmol/l) mean ± SD	1.59 ± 4.96	1.1 ± 0.33	1.03 ± 0.18	0.187
AST (U/L), median (IQR)	28 (17,38)	29 (22,37)	25 (15.75,46.25)	0.702
ALT (U/L), median (IQR)	24 (20,35)	25 (22,39)	23 (18,30.25)	0.597
STB (umol/L) mean ± SD	15.38 ± 6.94	15.97 ± 8.54	15.0 ± 5.67	0.434
eGFR (mL/min) mean ± SD	114.02 ± 12.42	116.3 ± 11.27	112.53 ± 12.97	0.095
Scr(mL/min) mean ± SD	78.58 ± 181	98.11 ± 38.92	65.14 ± 14.85	0.309
**Other risk factors**				
Cirrhosis (%)	16/137 (11.68)	10/56 (17.86)	6/81 (7.41)	0.061
Fatty liver (%)	48/137 (35.04)	19/56 (33.93)	29/81 (35.80)	0.821
Hypertension (%)	8/137 (5.84)	1/56 (1.79)	7/81 (8.64)	0.19
Diabetes (%)	7/137 (5.11)	3/56 (5.36)	4/81 (4.94)	1.00

HC, hypercholesterolemia; NHC, non-hypercholesterolemia; SD, standard deviation; IQR, interquartile range; ART, antiretroviral therapy, HDL, high density lipoprotein; LDL, low-density lipoprotein; AST, aspartate aminotransferase; ALT, alanine transaminase. Scr, Serum creatinine; eGFR, estimated glomerular filtration rate; HBsAg, hepatitis B surface antigen.

### 3.2 Virologic outcomes after TAF treatment

**FIGURE 2:**
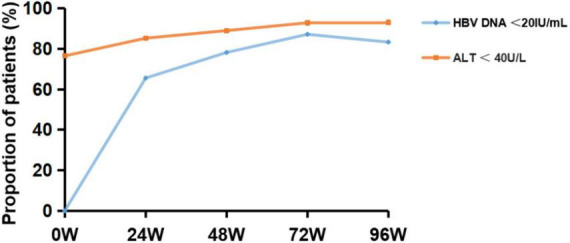
Virological response outcome before and after 24, 48, 72 and 96 weeks of TAF treatment in HBV patients. HBV-DNA, hepatitis B virus-deoxyribonucleic acid; ALT, alanine transaminase.

The proportion of patients with HBV DNA < 20 IU/mL was significantly higher after 24 W, 48 W, 72 W, and 96 W of TAF treatment, compared with baseline (65.57%, 78.21%, 87.18%, 83.33% vs. 18.11%, *P* < 0.001) (Figure 2). The proportion of patients with ALT < 40 U/L was significantly higher after 24 W, 48 W, 72W and 96 W of treatment, compared with baseline (85.27%, 89.02%, 92.86%, 93.10% vs. 76.52%, *P* < 0.05) (Figure 2). In addition, the proportion of HBeAg-negative patients increased to 51.85% and serum HBsAg levels gradually decreased to 3.21 ± 0.97 log IU/mL after 96W of TAF treatment.

### 3.3 Lipid profile during TAF treatment in total CHB patients

**FIGURE 3:**
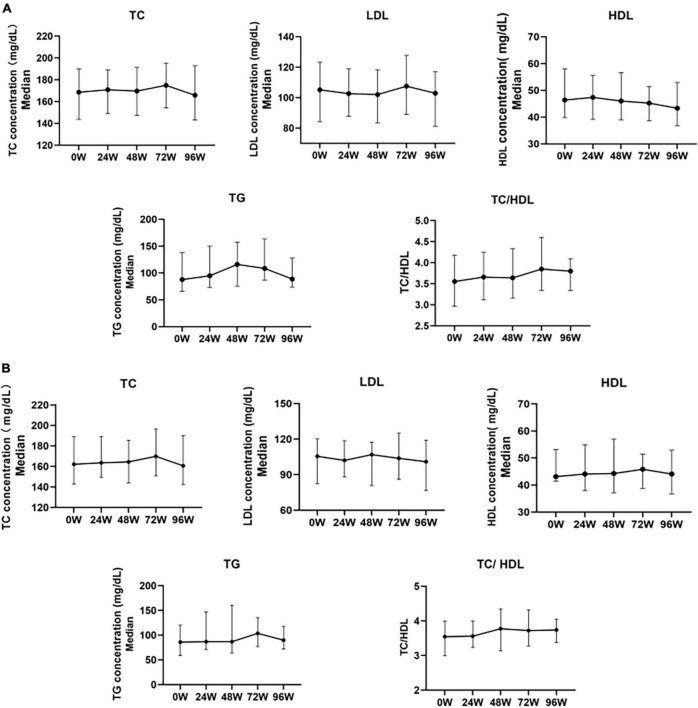
Changes in serum lipoproteins before and after 24, 48, 72, and 96 weeks of univariate analysis of TAF treatment in HBV patients. **(A)** Changes in Lipid Levels in Overall Patients After Treatment with TAF (0W: *n* = 137, 24W: *n* = 137, 48W: *n* = 89, 72W: *n* = 62, 96W: *n* = 60). **(B)** Changes in blood lipid profile in a small sample of HBV patients with a complete follow-up of 96 weeks after treatment with TAF (*n* = 36). TC, Total Cholesterol; LDL, Low Density Lipoprotein; HDL, High Density Lipoprotein; TG, Triglyceride. The data were shown as the median (IQR).

Cross-sectional results showed that TC and LDL-c levels gradually increased during treatment with TAF, increased to 174.94 mg/dL (+6.38mg/dL, *P* > 0.05) and 107.47 mg/dL (+2.32 mg/dL, *P* > 0.05) at 72W respectively, gradually decreased after 72 W, and slightly decreased relative to the baseline at 96 W, but there was no statistical difference (*P* > 0.05). HDL-c levels did not change significantly during 96 W of treatment with TAF (*P* > 0.05). TG levels gradually increased before 48 W, increased to 116.07 mg/dL (+28.35mg/dL, *P* = 0.009)at 48 W, and then gradually decreased after 48 W. There was no significant change relative to the baseline at 96 W (+0.90mg/dL, *P* > 0.05). TC/HDL levels slightly increased during the use of TAF, and increased to 3.80 by 96 W (+0.24, *P* > 0.05) (Figure 3A).

In the 96W cohort study (*N* = 36), the changes in TC, LDL-c, HDL-c, TG, and TC/HDL ratio after TAF treatment were generally consistent with the overall lipid changes in the above cross-sections (Figure 3B).

### 3.4 Lipid profile during TAF treatment in CHB patients combined with NHC and HC

**FIGURE 4:**
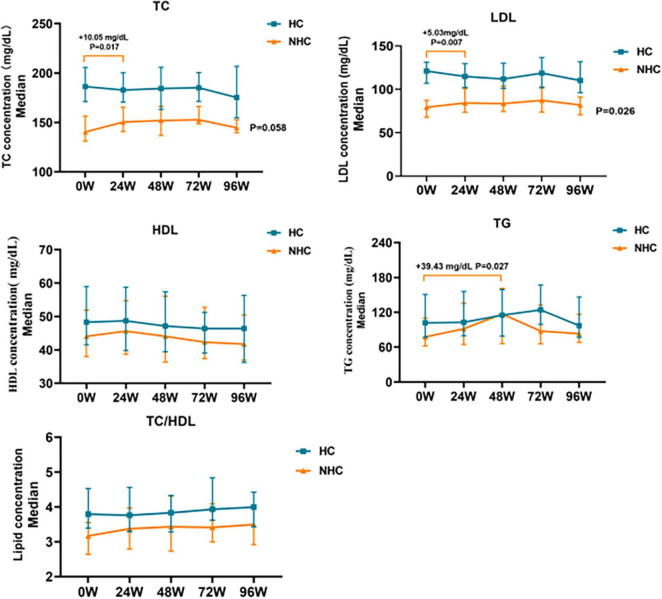
Changes in relative baseline lipid levels after TAF treatment in HBV patients with NHC (*n* = 56) and HC (*n* = 81). NHC, non-hypercholesterolemia; HC, hypercholesterolemia; TC, Total Cholesterol; LDL, Low Density Lipoprotein; HDL, High Density Lipoprotein; TG, Triglyceride. The data were shown as the median (IQR).

TC and LDL levels in non-hypercholesterolemic CHB patients increased significantly at 24 W (*P* < 0.05). Serum TC and LDL levels increased to 150.39 mg/dL (+10.05 mg/dL, *P* = 0.017) and 84.28 mg/dL (+5.03 mg/dL, *P* = 0.007) respectively, and then gradually declined after 24 W. There was no significant change at 96W compared with baseline levels (+4.25 mg/dL and +2.71 mg/dL, *P* > 0.05). TG levels increased to 116.95mg/dL at 48W, which was a significant increase compared with the baseline (+39.43 mg/dL, P = 0.027), gradually decreased after 48W. There was no significant change at 96W compared with baseline levels (+5.76 mg/dL, *P* > 0.05) as showed in Figure 4.

During treatment with TAF in patients combined with hypercholesterolemia, TC and LDL levels gradually decreased before 72 W and gradually increased after 72 W, but none of them were statistically different (*P* > 0.05). TG levels did not change significantly during treatment with TAF (*P* > 0.05), as shown in Figure 4.

Regardless of whether hypercholesterolemia was combined or not, HDL levels gradually decreased in CHB patients treated with TAF, TC/HDL ratio slightly increased in CHB patients with TAF, increased to 4.0 (+0.20, *P* > 0.05) and 3.49 (+0.33, *P* > 0.05) by 96 W, but none of them were statistically different (*P* > 0.05), as shown in Figure 4.

### 3.5 Correlation and multiple linear regression analysis of TAF-related lipid level changes with AST, ALT, and CAP values

Lipid levels and lipid ratios were not significantly correlated with age and HBsAg levels, while they were positively correlated with CAP values for fatty liver (*P* < 0.05) in patients after 24 weeks of TAF treatment. In addition, the levels of TC, LDL, and HDL were significantly and positively correlated with AST and ALT after TAF treatment (*P* < 0.05), as shown in Table 2. Therefore, a multiple regression analysis of the influencing factors was further performed.

**TABLE 2:** Correlation of lipid levels with age, HBsAg level, and CAP value after TAF treatment.

Prediction variable	Age		HBsAg		CAP		ALT		AST	
	r	P	r	P	r	P	r	P	r	P
TC	0.081	0.354	0.025	0.796	0.207	0.058	0.282	0.001	0.233	0.008
LDL	0.118	0.179	0.002	0.980	0.240	0.027	0.237	0.007	0.172	0.053
HDL	0.096	0.276	0.037	0.706	0.229	0.036	0.231	0.009	0.283	0.001
TG	0.087	0.321	0.083	0.391	0.404	<0.001	0.014	0.880	0.058	0.513
TC/HDL	0.02	0.82	0.028	0.775	0.339	0.002	0.016	0.855	0.104	0.246

HBsAg, hepatitis B surface antigen; CAP, controlled attenuation parameter; ALT, alanine transaminase; AST, aspartate aminotransferase.

Linear regression analysis with CAP value as the dependent variable showed that CAP value was the influencing factor of lipid levels or ratios after TAF treatment(*P* < 0.05), as shown in Table 3. Linear regression analysis with AST and ALT as the dependent variables showed that AST and ALT were the influencing factors of cholesterol (TC, LDL, and HDL) levels (Table 4).

**TABLE 3:** Multiple linear regression analysis of lipid levels and CAP values after TAF treatment.

Prediction variable	B	SE	β	t	P
**TAF 24W**					
TC	0.003	0.002	0.207	1.924	0.058
LDL	0.003	0.001	0.24	2.252	0.027
HDL	-0.001	0.001	-0.229	-2.128	0.036
TG	0.008	0.002	0.404	4.024	0
TC/HDL	0.007	0.002	0.339	3.278	0.002
**TAF 48W**					
TC	0.003	0.002	0.193	1.421	0.161
LDL	0.001	0.002	0.104	0.753	0.455
HDL	-0.002	0.001	-0.284	-2.132	0.038
TG	0.008	0.005	0.222	1.639	0.107
TC/HDL	0.008	0.003	0.409	3.235	0.002
**TAF 72W**					
TC	0.008	0.003	0.476	2.867	0.008
LDL	0.007	0.003	0.446	2.638	0.013
HDL	0.000	0.001	-0.006	-0.029	0.977
TG	0.006	0.005	0.243	1.324	0.196
TC/HDL	0.007	0.004	0.354	2.002	0.055
**TAF 96W**					
TC	0.011	0.003	0.638	3.614	0.002
LDL	0.01	0.003	0.676	3.997	0.001
HDL	0.000	0.001	0.011	0.046	0.964
TG	0.009	0.005	0.355	1.655	0.114
TC/HDL	0.01	0.004	0.475	2.351	0.03

**TABLE 4:** Multiple linear regression analysis of lipid levels and ALT and AST after TAF treatment.

Prediction variable	ALT					AST				
	B	SE	β	t	P	B	SE	β	t	P
**TAF24W**										
TC	0.004	0.001	0.282	3.3	0.001	0.005	0.002	0.233	2.69	0.008
LDL	0.003	0.001	0.237	2.723	0.007	0.003	0.002	0.172	1.954	0.053
HDL	0.001	0	0.231	2.646	0.009	0.002	0.001	0.283	3.282	0.001
TG	0.000	0.001	-0.014	-0.152	0.88	-0.001	0.002	-0.058	-0.657	0.513
TC/HDL	0.000	0.001	-0.016	-0.183	0.855	-0.003	0.002	-0.104	-1.166	0.246
**TAF48W**										
TC	0.004	0.001	0.339	3.221	0.002	0.008	0.002	0.352	3.361	0.001
LDL	0.003	0.001	0.292	2.73	0.008	0.006	0.002	0.302	2.836	0.006
HDL	0.001	0	0.265	2.463	0.016	0.003	0.001	0.343	3.268	0.002
TG	0.000	0.002	-0.01	-0.09	0.928	-0.001	0.004	-0.035	-0.31	0.757
TC/HDL	0.00	0.001	-0.01	-0.091	0.927	-0.001	0.003	-0.057	-0.51	0.611

ALT, alanine transaminase; AST, aspartate aminotransferase.

### 3.6 Lipid profiles during TAF treatment in CHB patients with fatty liver and non-fatty liver disease

Because the CAP value of fatty liver is an effect factor in lipid levels, our subgroup analysis found that TC and LDL-c levels gradually increased at 96 W in CHB patients with fatty liver treated with TAF, which increased to 196.7 mg/dL (+28.76 mg/dL, *P* = 0.06) and 127.2 mg/dL (+22.58 mg/dL, *P* > 0.05), respectively. TG levels gradually increased to 146.63 mg/dL (+37.67 mg/dL, *P* = 0.057) at 48 W, and then gradually decreased, and there was no significant change at 96 W compared to baseline. There was no significant change in HDL-c levels during TAF treatment (*P* > 0.05).

**FIGURE 5:**
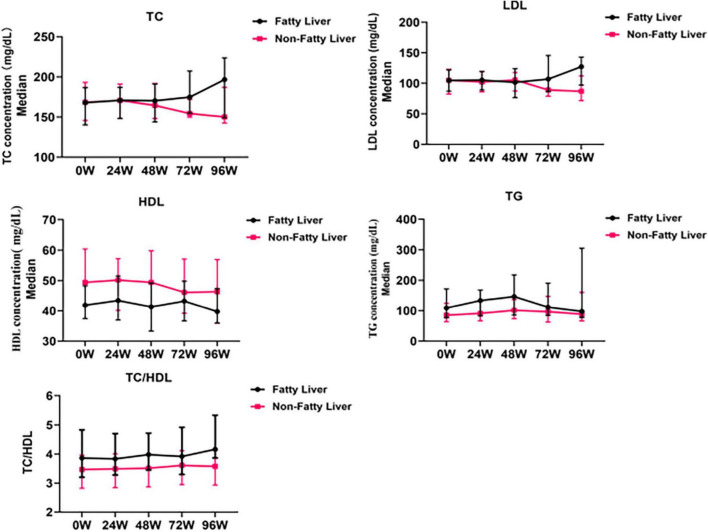
Changes in the lipid levels during TAF treatment in HBV patients with fatty liver (n = 48) and non-fatty liver (*n* = 89). TC, Total Cholesterol; LDL, Low Density Lipoprotein; HDL, High Density Lipoprotein; TG, Triglyceride. The data were shown as median (IQR).

None of the lipid profiles of CHB patients with non-fatty liver disease also changed significantly (*P* > 0.05) during treatment with TAF (Figure 5).

Regardless of whether fatty liver was combined or not, the TC/HDL ratio slightly increased in CHB patients treated with TAF, increased to 4.16 (+0.30, *P* > 0.05) and 3.58 (+0.11, *P* > 0.05) at 96 W (Figure 5).

## 4 Discussion

With the wide application of TAF, the lipid safety of TAF is a great clinical concern (17). In this study, through cross-sectional and cohort studies, the lipid profiles were demonstrated in CHB patients treated with TAF, in patients including hypercholesterolemia and fatty liver disease.

TAF is the first-line antiviral drug treatment for CHB patients, with potent antiviral effects (18). The results of our study also showed high complete viral suppression rate and ALT normalization rate during TAF treatment, which were consistent with the previous clinical studies (19, 20)

Previous studies have found that a greater increase in TC levels was observed in patients with CHB receiving TAF compared to those receiving ETV (16). In this study, it was firstly found that TAF had low effect on lipids in a cross-sectional study, and furthermore, a long-term cohort study was conducted to clarify that the use of TAF in CHB patients for 96W was safe.

The results of our study showed that a gradual decrease in LDL-c levels during TAF treatment. TC, HDL-c and TG levels increased at 24W, then gradually decreased after 24W and were lower than the baseline at 96W. None of the lipid changes showed significant differences. Similar studies have also shown a slight increase in lipids in HIV patients within one year of antiretroviral therapy, and lipid changes were static and not significantly different after 48 W, and TAF may not cause deterioration in the lipid profile of the subjects (11, 21). In our cohort study, the results were generally consistent with the lipid changes in the preceding cross-sections. In stratified analysis, CHB patients combined with non-hypercholesterolemic showed no significant changes in lipid profiles, especially for TC and LDL-c, after 96W of TAF treatment. Thus, lipid dynamics in CHB patients were safe during 96W of TAF treatment.

In our study, insignificant changes were found in lipid levels in CHB patients with TAF for 96W. Also, studies such as Chan et al. suggested that the effect of TAF on lipid levels was not clinically significant (22). However, a retrospective multicentering study showed significantly elevated levels of hypercholesterolemia, HDL-C, LDL-C, non-HDL-C, and oxidized LDL in CHB patients who switched from TDF to TAF, with an increased incidence of dyslipidemia (from 33% to 39%) (23). Therefore, the conclusions of this study need to be confirmed by longer-term studies.

Patients with CHB combined with hypercholesterolemia showed a trend towards lower TC and LDL-C levels with TAF for 96W, but there was no significant difference, and no significant change in TG levels. Similar findings also found that in HIV patients diagnosed with hypercholesterolemia at baseline, HDL-c was elevated after TAF treatment, whereas TC and LDL-levels did not change significantly during TAF treatment (24, 25).

The TC/HDL ratio is recognized as an important cumulative indicator of cardiovascular risk (26, 27). The results of this study showed a slight increase in TC/HDL ratio after TAF treatment. More importantly, the TC/HDL ratio of patients with hypercholesterolemia did not change significantly after TAF treatment. Other studies have shown that compared with baseline, no significant increase in overall ASCVD risk or any ASCVD risk category was observed during the 96-week treatment period, which was similar in the TAF and TDF groups (28). Hong’s study also showed that the association between long-term TDF or TAF therapy and changes in lipid profiles may influence or increase the impact on cardiovascular risk to some extent, if at all, but is not one of the main determinants (29). Therefore, TAF in patients with dyslipidemia may not increase the risk of cardiovascular disease. However, longer-term follow-up of lipid profiles in patients using TAF is necessary to further assess the impact on CVD risk.

Through multiple regression analysis, our study confirmed that baseline ALT and AST levels and CAP values were related to lipid safety during TAF treatment. Previous studies have shown that aminotransferase levels were significantly associated with hepatic inflammation caused by HBV viral load (30, 31). In addition, there is a negative correlation between hepatic steatosis and HBV activity (32), and it has been shown that accumulation of saturated fatty acids initiates an immune response that inhibits HBV replication; accelerates HBsAg and HBV-DNA clearance by increasing apoptosis of HBV-infected cells (33). It is hypothesized that TAF treatment reduces HBV viral load, promotes hepatic recovery, reduces aminotransferase levels, and further restores lipid metabolism.

CAP measurements can reflect the degree of hepatic steatosis (34). Based on this, our subgroup analysis based on whether CHB patients had comorbid fatty liver or not, which revealed that CHB patients with fatty liver did not have significant changes in their lipid profile after TAF treatment, and the results of our study did not show a significant effect on TAF-related lipid change in patients with fatty liver, which requires further observation.

The studies of HIV-infected patients have found an association between TAF use and hepatic steatosis (35). Relatively few studies have been conducted on whether TAF use exacerbates steatosis in patients with CHB, however, some research indicated TAF may increase patient weight (36). Therefore, CHB combined with fatty liver may also be a risk factor for TAF-related safety, and further randomized controlled and large-sample trials are needed to validate the changes in lipid profile after TAF treatment, to assess the risk of cardiovascular disease for the population with fatty liver disease, and to provide a rational basis for the use of antiviral therapy in the fatty liver population.

In this study, we observed the dynamic changes in lipids after antiviral therapy in patients with CHB who were initially treated with TAF. However, there’s limitation in our study, the sample size of our study was small, and lack of other antiviral drug treatments as control group. In the future, a larger sample size, well controlled study should be designed and conducted to improve the rigor and objectivity of the study.

## 5 Conclusion

TAF treatment had a low effect on the lipid profile of CHB patients over the course of 96 weeks, and it was safe in patients combined with fatty liver and hypercholesterolemia. Further effects of TAF use on lipids and the potential role on cardiovascular disease risk require more prolonged observation as well as mechanistic studies.

## Data availability statement

The raw data supporting the conclusions of this article will be made available by the authors, without undue reservation.

## Ethics statement

The studies involving humans were approved by the First Affiliated Hospital of Xi’an Jiaotong University Ethics Committee. The studies were conducted in accordance with the local legislation and institutional requirements. The participants provided their written informed consent to participate in this study. Written informed consent was obtained from the individual(s) for the publication of any potentially identifiable images or data included in this article.

## Author contributions

WZ: Data curation, Formal analysis, Writing–original draft, Writing–review and editing. YL: Methodology, Writing–review and editing. MZ: Formal analysis, Writing–review and editing. ZC: Formal analysis, Writing–review and editing. ZQ: Data curation, Writing–review and editing. YL: Formal analysis, Writing–review and editing. MW: Data curation, Writing–review and editing. WW: Data curation, Formal analysis, Writing–review and editing. YC: Data curation, Writing–review and editing. LS: Data curation, Investigation, Writing–review and editing. JL: Data curation, Formal analysis, Writing–review and editing. FY: Formal analysis, Writing–review and editing.
